# Living with Chronic Illness Scale: International validation through the classic test theory and Rasch analysis among Spanish‐speaking populations with long‐term conditions

**DOI:** 10.1111/hex.13351

**Published:** 2021-09-07

**Authors:** Carmen Rodriguez‐Blazquez, Maria João Forjaz, Alba Ayala, Mari Carmen Portillo, Leire Ambrosio, EC‐PC Validation Group

**Affiliations:** ^1^ National Epidemiology Centre, Carlos III Institute of Health Madrid Spain; ^2^ Network Center for Biomedical Research in Neurodegenerative Diseases (CIBERNED) Madrid Spain; ^3^ Health Service Research Network on Chronic Diseases (REDISSEC); ^4^ University Carlos III of Madrid Madrid Spain; ^5^ School of Health Sciences, NIHR ARC Wessex University of Southampton Southampton United Kingdom

**Keywords:** classic test theory, living with, long‐term condition, person‐centred tool, psychometric properties, Rasch analysis, Spanish

## Abstract

**Background:**

The Living with Chronic Illness (LW‐CI) Scale is a comprehensive patient‐reported outcome measure that evaluates the complex process of living with long‐term conditions.

**Objective:**

This study aimed to analyse the psychometric properties of the LW‐CI scale according to the classic test theory and the Rasch model among individuals living with different long‐term conditions.

**Design:**

This was an observational, international and cross‐sectional study.

**Methods:**

A total of 2753 people from six Spanish‐speaking countries living with type 2 diabetes mellitus, chronic obstructive pulmonary disease, chronic heart failure, Parkinson's disease, hypertension and osteoarthritis were included. The acceptability, internal consistency and validity of the LW‐CI scale were analysed using the classical test theory, and fit to the model, unidimensionality, person separation index, item local independency and differential item functioning were analysed using the Rasch model.

**Results:**

Cronbach's *α* for the LW‐CI scale was .91, and correlation values for all domains of the LW‐CI scale ranged from .62 to .68, except for Domain 1, which showed correlation coefficients less than .30. The LW‐CI domains showed a good fit to the Rasch model, with unidimensionality, item local independency and moderate reliability providing scores in a true interval scale. Except for two items, the LW‐CI scale was free from bias by long‐term condition type.

**Discussion:**

After some adjustments, the LW‐CI scale is a reliable and valid measure showing a good fit to the Rasch model and is ready for use in research and clinical practice. Future implementation studies are suggested.

**Patient and Public Contribution:**

Patient and public involvement was conducted before this validation study ‐ in the pilot study phase.

## 1 INTRODUCTION

Nowadays, long‐term conditions (LTCs) are the leading causes of disability and costs worldwide.^1^ It is estimated that, by 2030, LTCs will account for three quarters of deaths globally, with considerable social and economic impact due to the exorbitant costs to the system.^2^, ^3^ In particular, cardiovascular and respiratory diseases, as well as diabetes, are the LTCs that account for the most deaths woldwide.^1^

Based on previous studies, living with an LTC is defined as a complex, dynamic, cyclical and multidimensional process that involves five different attributes, namely, acceptance, coping, self‐management, integration and adjustment.^4^ Living with an LTC is influenced by social and personal factors; therefore, living with an LTC is a unique, individual and unrepeatable process.^4^, ^5^ Health and social professionals need to have an in‐deep understanding of what it means to live with an LTC from the person's perspective. They should focus on the psychosocial and spiritual areas of the person and not just on the condition as has been done thus far.^1^, ^4^, ^6^ In this sense, use of patient‐reported outcome measures (PROMs) is paramount to evaluate how the person is living with his/her condition.^7^, ^8^ Consequently, an interdisciplinary team could design individualized and comprehensive care pathways and recommendations to achieve positive living with an LTC as a final desired target.^7^, ^8^ Currently, most relevant available PROMs in clinical practice and research evaluate specific aspects of the LTC (e.g., stage, symptoms or severity) or outcomes of the process of living with LTCs (e.g., quality of life, satisfaction with life or well‐being).^9^ The Long‐Term Conditions Questionnaire^10^ is the only PROM that potentially evaluates how a person is living with/managing an LTC. However, this scale seems to evaluate quality‐of‐life outcomes and does not tackle items related to acceptance, which is an essential attribute when living with an LTC.^4^ Inevitably, this left a gap regarding person‐centred measures that can evaluate the process of living with an LTC. To fill this gap and based on previous empirical studies^11^, ^12^ and conceptual works,^4^, ^13^ the Living with Chronic Illness (LW‐CI) Scale was designed.^14^

The LW‐CI scale is a comprehensive PROM that evaluates the complex process of living with an LTC.^14^, ^15^ It is a 26‐item self‐reported scale with direct applicability in people living with LTCs. It was originally designed for Spanish‐speaking populations living with an LTC.^15^ The LW‐CI scale has been previously published for Spanish‐speaking populations in a pilot study carried out among patients living with different LTCs.^15^ To date, the LW‐CI scale has been tested separately in several LTCs, namely, Parkinson's disease (PD),^16^, ^17^ osteoarthritis,^18^ chronic heart failure,^19^ type 2 diabetes mellitus (T2DM)^20^ and chronic obstructive pulmonary disease (COPD).^21^ The results from each validation study showed that the LW‐CI scale is a feasible, reliable and valid measure to evaluate separately the process of living with an LTC in a Spanish‐speaking population. According to those validation studies,^16^, ^17^, ^18^, ^19^, ^20^, ^21^ potential modifications were proposed to achieve a better version of the LW‐CI scale for each LTC in particular, such as simplifying the response scale, deleting some items or redesigning the domains. However, could those previous specific‐disease validation results be extended across LTC populations? There is an opportunity to test the psychometric properties of the LW‐CI scale in a sample representing diverse chronic health conditions to validate the measurement properties across persons living with different LTCs.

All LW‐CI scale validation studies were conducted using a classical test theory (CTT) approach to evaluate reliability, validity and sensitivity to change along with acceptability and other parameters, mostly based on correlations and mean difference analyses. Only the LW‐CI for people with PD was additionally tested using Rash measurement analysis.^17^ The application of the Rasch model,^22^ one of the most used applications of the item response theory, combined with the classic test theory approach is recommended for evaluating PROM.^23^, ^24^, ^25^ The Rasch model^22^ completes the information provided by the CTT because it provides additional and relevant information about the measurement properties of a scale such as unidimensionality and differential item functioning (DIF) by individual groups including LTC type. In addition, it also allows for the calculation of scores on a linear scale.^26^

Therefore, the aim of this study is to analyse the psychometric properties of the LW‐CI scale according to the classic test theory and the Rasch model among people living with different LTCs. Suggestions for modification of the LW‐CI scale are presented accordingly.

## 2 METHODS

### 2.1 Design

This was an international and cross‐sectional study. This study is part of a multicentric and multidisciplinary research programme led by nurses (ReNACE Programme; https://www.unav.edu/web/programa-renace/proyectos) aimed at achieving an in‐depth understanding of the complex process of living with an LTC from the persons' and family/carers' perspectives through the development of individualized interventions and comprehensive PROM.^11^, ^12^ In particular, this study has the general aim of achieving a unique and standardized international Spanish‐speaking self‐reported scale to evaluate how the person is living with his/her LTC in several Spanish‐speaking countries.

### 2.2 Setting

Participants were recruited from different healthcare systems from six Spanish‐speaking countries, namely, Spain, Cuba, Argentina, Ecuador, Mexico and Colombia. More concretely, private and public primary and specialized healthcare service‐attending outpatients with LTCs were included. Additionally, individuals living with LTCs from the community were also approached, mainly from LTC organisations in Spain, such as the Parkinson's Disease Association.

### 2.3 Participants

Sampling of consecutive cases^27^, ^28^ was applied to select participants. The sample was composed of individuals living with different LTCs, namely, T2DM, COPD, chronic heart failure (HF), PD, hypertension and osteoarthritis. The following criteria were applied: (1) diagnosed with an LTC by a general practitioner or consultant (T2DM, COPD, HF, PD, hypertension and osteoarthritis); (2) adult patient (≥18 years); (3) able to read, understand and answer written questionnaires; (4) native Spanish‐speaking person; and (5) able to provide written informed consent. The applied exclusion criteria were as follows: (1) presence of cognitive deterioration or psychiatric disorders, or any other disorder that could interfere with or impede the appropriate development of the study (e.g., blindness); (2) hospitalized patients; and (3) patients not fulfilling the established inclusion criteria.

According to international criteria, the sample size was estimated to fulfil the rule of 10 participants per item,^29^ which exceeds the minimum of 100 subjects required for CTT. Therefore, considering that the LW‐CI scale is a 26‐item scale, the minimum sample size estimated was 260 participants per condition, aiming for a total of 1560 participants.

### 2.4 Assessments

Sociodemographic data, such as age, gender, marital status, employment situation and educational level, were collected. In addition, LTC‐related information was collected, namely, age at disease and disease duration. In addition to sociodemographic and LTC‐related data, the Spanish version of the following self‐reported validated PROMs was used:

1. LW‐CI scale^15^: The LW‐CI scale is a self‐reported scale that evaluates the complex process of living with an LTC. The LW‐CI scale is a 26‐item scale grouped into five domains: Domain 1: Acceptance (4 items); Domain 2: Coping (7 items); Domain 3: Self‐management (4 items); Domain 4: Integration (5 items); and Domain 5: Adjustment (6 items).^15^ All items are answered using a 5‐point Likert scale from never or nothing (0) to always or a lot (4), except for Domain 1: Acceptance, which is reversely scored from never or nothing (4) to always or a lot (0). The LW‐CI scale has a total score value ranging from 0 points, indicating negative living with the condition, to 104 points, reflecting positive living with the condition.
2. Duke‐UNC Functional Social Support Questionnaire (DUFSS)^30^: The DUFSS is a self‐reported measure that comprises 11 items evaluating diverse dimensions of social support such as confidant, affective and instrumental support. The score for each item varies from 1 (*much less than I would like*) to 5 (*as much as I would like*). The total score ranges from 11 (lowest level of support ‘*much less than I would like*’) to 55 (highest level of support ‘*as much as I like*’). According to the Spanish validation study,^31^ the DUFSS presented adequate psychometric properties, showing a Cronbach's *α* value of .9 and satisfactory construct validity.^30^, ^31^
3. Modified version of the Satisfaction with Life Scale (SLS‐6): Originally, this is a 7‐item self‐reported questionnaire.^32^ For this study, the modified version of the SLS‐6 was used because the original version and, in particular, one of the items were specific for a student population.^33^ In this way, a modified version with a 6‐item scale was used to evaluate the degree of overall satisfaction with life and regarding six areas: whole life, physical, psychological well‐being, social relations, leisure and financial situation. Each item was rated on a 0 (*totally unsatisfied with life*) to 10 (*totally satisfied with life*)‐point Likert scale. According to the Spanish validation study,^33^ the SLS‐6 presented satisfactory psychometric properties, with a Cronbach's *α* of .8 and internal validity values ranging from .4 to .7.^32^, ^33^
4. Patient‐Based Global Impression of Severity Scale (PGIS): This is an adaptation of the clinical global impression of severity^34^ adapted for patients as a self‐reported scale.^35^ It is a global index that may be used to assess self‐perception of disease severity of the person living with a disease. The PGIS is a 6‐point Likert scale, ranging from 0 (*not ill at all*) to 5 (*extremely ill*). It has excellent construct validity and has been widely used in studies of chronic diseases.^36^

### 2.5 Data collection

For people living with PD, data were collected between January and June 2015, and for people living with T2DM, COPD, HF, hypertension and osteoarthritis, data were collected between November 2018 and May 2019 among the different healthcare centres and community settings of six Spanish‐speaking countries. Although data collection was performed at different times, a detailed and standardized protocol was used to ensure data collection homogeneity and reduce possible errors during the process.^37^ According to the established protocol, potential participants were approached through a health professional (nurse or physician) or member of the research group. An invitation letter and a participant information sheet as well as verbal information were provided about the study. Participants who agreed to participate signed the consent form and were asked to complete the sociodemographic and LTC‐related data as well as PROM when they agreed with his/her health professional or researcher. Hence, participants completed the scales during consultations with the GP, specialized clinician, nurse specialist or primary care nurse. Participants who required help to complete the scales (e.g., due to vision problems) were assisted by a researcher. Once the participants finished answering the scales, a researcher reviewed the answers to identify possible missing data. Hence, no missing data were expected.

### 2.6 Ethical aspects

The study was approved by the research ethics committee of the principal investigator centre (reference numbers: 071/2014 and 2017.099) and all the included centres. This validation study was adjusted to the principles outlined in the Declaration of Helsinki (1964) of Law 14/2007 on Biomedical Research and Law 15/1999 on the Protection of Personal Data. All participants provided their signed consent to participate voluntarily after receiving verbal and written information related to the study. Signed consent was provided in front of the health professional or a member of the research group before completing the surveys.

### 2.7 Data analysis

Descriptive statistics, namely, central tendency measures and proportions, were used to determine the sociodemographic and LTC characteristics of the participants. The main data were ordinal or did not fit a normal distribution. Therefore, nonparametric statistics were used.

According to the CTT, the following psychometric attributes were analysed:

1. Feasibility and acceptability: The quality of the data was considered satisfactory if 95% of the data were computable. The limit for missing data was less than 5%,^38^ and the mean, median and standard deviation (SD) were estimated to be roughly equivalent (≤10% maximum punctuation).^39^ Floor and ceiling effects were deemed acceptable if they were less than 15%,^39^ and the skewness was expected to be between −1 and +1.^40^
2. Reliability: Internal consistency was tested by Cronbach's *α* coefficient (criterion value ≥ 0.70),^41^ item‐total correlation (corrected for overlap; criterion value, *r*_s_ ≥ .30),^23^ inter‐item correlation (criterion value, *r* ≥ .20 and *r* ≤ .75)^38^, ^42^ and item homogeneity (criterion value > 0.30).^43^
3. Construct validity: For convergent validity, a moderate (*r*_s_ ≥ .35–.50) or strong relationship (*r*_s_ > .50)^44^, ^45^, ^46^ was hypothesized between the LW‐CI scale and DUFSS and SLS‐6, according to previous studies. A weak (*r*_s_ = .20–.34) or insignificant (*r*_s_ = .00–.19) relationship with age at diagnosis and disease duration was also hypothesized. These hypotheses were established to corroborate previous LW‐CI scale validation study results in specific LTCs.^16^, ^18^, ^19^, ^20^, ^21^ Spearman's rank correlation coefficients were obtained for this purpose. Internal validity, defined as the intercorrelations between the LW‐CI scale dimensions (standard, *r*_s_ = .30–.70),^40^, ^47^ was determined. For known groups' validity, differences in LW‐CI scale scores in the participants grouped by gender and PGIS scores were analysed.^47^, ^48^ Mann–Whitney and Kruskal–Wallis tests with Bonferroni corrections were used for group comparisons.

For the Rasch model, the following measurement properties were analysed: fit to the Rasch model, reliability (person separation index [PSI]), adequacy of the response scale, item local independence and unidimensionality. In addition, DIF was analysed by the following factors: sex, age groups (70 years or younger vs. older than 70 years), disease duration (categorized by the median; up to 6 vs. 7+ years) and LTC type; DIF by country was not possible due to an unequal distribution of data from different countries, precluding comparisons.

According to the Rasch model, the response to a certain item is a function of the person's ability (or experienced level of the construct) and the item's difficulty (or the measured level of construct by that item), expressed in logits.^22^ Fit to the Rasch model is observed when there is a nonsignificant *χ* ^2^ difference between the data and the Rasch model, with Bonferroni correction by number of items.^26^ In addition, residuals should follow a normal distribution (mean of 0; SD of 1) and fall within the ±2.5 range. Large sample sizes might result in a high statistical power to detect small model deviations and unnecessary modifications. Therefore, for Rasch analysis, we included a random sample of 300 individuals.^49^

Reliability is expressed by the PSI, interpreted similarly to Cronbach's *α*. A threshold is the point of equal response probability between two adjacent categories. Its order is analysed and in the case of disordered thresholds, adjacent categories are collapsed. For items to be locally independent, we expect low correlations (<.30 of the average correlation) between item residuals.^50^ Unidimensionality is measured through a principal component of the residuals, and person estimates are compared using *t*‐tests. A lower bound of the associated binomial 95% confidence interval (CI) less than 5% indicates unidimensionality.^51^, ^52^ For DIF, analyses of variance (ANOVA) with Bonferroni correlation are conducted for all factors.^53^ When several items present significant DIF by a certain factor, a top–down purification procedure is followed by creating two groups of items, with or without DIF, and comparing the estimates in an ANOVA to see if DIF remains.^54^ Model modifications were evaluated iteratively.

SPSS 22.0 (IBM Corp.) was used for all CTT analyses, and RUMM2030 was used for Rasch analysis.

## 3 RESULTS

A total of 2753 people living with different LTCs were included in the sample. Osteoarthritis presented the lowest sample size (*n* = 291), and T2DM presented the highest sample size (*n* = 582). Demographic information is shown in Table 1. The age range was 20–98 years, with a mean age of 68.21 years (SD: 12.21 years). More than half of the sample was female (*n* = 1441, 52.3%), was married (*n* = 1555, 56.5%) and had basic/primary education levels (*n* = 1596, 58.1%). The employment status of the sample was mainly distributed between been retired (*n* = 934, 34%) and working as housekeeper (*n* = 827, 30.1%). All of the participants had at least one LTC, with a duration of 9.80 years (SD: 8.65; range: 0–67 years) and a mean age at diagnosis of 58.37 years (SD: ±13; range: 3–91 years).

**Table 1:** Participant characteristics (*n* = 2753)

Demographic variable	Response option	*N* (%)
Country	Argentina	60 (2.2)
	Colombia	1329 (48.3)
	Cuba	50 (1.8)
	Ecuador	60 (2.2)
	Mexico	53 (1.9)
	Spain	1201 (43.6)
Long‐term condition	Type 2 diabetes mellitus	582 (21.1)
	Chronic obstructive pulmonary disease	612 (22.2)
	Chronic heart failure	603 (21.9)
	Parkinson's disease	324 (11.8)
	Hypertension	341 (12.4)
	Osteoarthritis	291 (10.6)
Gender	Male	1312 (47.7)
	Female	1441 (52.3)
Marital status	Single	316 (11.5)
	Married	1555 (56.5)
	Widower	612 (22.3)
	Separated/divorced	261 (9.5)
	Others	6 (0.2)
Employment situation	Active working	501 (18.2)
	Housekeeper	827 (30.1)
	Retired	937 (34)
	Other	487 (17.7)
Educational level	Basic/primary studies	1596 (58.1)
	Secondary studies	709 (25.8)
	University studies	359 (13.1)
	Others	83 (3)
	Range	Mean (standard deviation)
Age	20–98	68.21 (12.21)
Age at diagnosis	3–91	58.37 (13.00)
Long‐term condition duration	0–67	9.80 (8.65)

### 3.1 CTT analysis

The results related to feasibility and acceptability showed that the LW‐CI scale was fully completed by 2738 participants, with 99.46% of the data computable. Levels of missing data were low and broadly uniform across domains, ranging from 0 missing data (Domain 1: Acceptance) to 6 (Domain 5: Adjustment). The floor effect was absent in all domains and in the total score, whereas Domains 1: Acceptance and 4: Integration showed ceiling effects (19.1% and 15.6%, respectively). For the five domains and the LW‐CI total scale, the difference between the mean and the median was less than 10%, the theoretical and observed ranges were coincident and the skewness values were between −1 and +1. Table 2 presents further feasibility and acceptability results.

**Table 2:** Feasibility, acceptability and reliability of the LW‐CI scale

	LW‐CI scale					
	Domain 1: Acceptance	Domain 2: Coping	Domain 3: Self‐management	Domain: 4 Integration	Domain 5: Adjustment	Total
Missing data/fully computable data	0/2753	2/2751	5/2748	2/2751	6/2747	15/2738
Mean	10.66	18.54	10.62	14.69	14.99	69.52
Median	11	19	11	15	15	70
Standard deviation	4.40	6.07	3.72	4.04	5.94	18.37
Observed range	0‐16	0‐28	0‐16	0‐20	0‐24	0‐104
Floor effect (%)	2.4	0.5	0.4	0.2	0.5	0.1
Ceiling effect (%)	19.1	6.2	12.9	15.6	12.6	1.6
Skewness	−0.59	−0.43	−0.36	−0.66	−0.17	−0.21
Cronbach's *α*	0.87	0.81	0.76	0.79	0.85	0.91
Item‐total correlation	0.618–0.80	0.418–0.65	0.488–0.60	0.388–0.67	0.498–0.68	–
Inter‐item correlation	0.48–0.73	0.238–0.53	0.308–0.55	0.208–0.62	0.298–0.61	–
Item homogeneity	0.62	0.38	0.44	0.42	0.48	–

Abbreviation: LW‐CI scale, Living with Chronic Illness Scale.

The results related to internal consistency showed that Cronbach's *α* for the LW‐CI scale was .91 and that for domains ranged between .76 (Domain 3: Self‐management) and .87 (Domain 1: Acceptance). All domains reached the present .30 threshold value for item‐total correlation, and item homogeneity index values were higher than 0.30 for all domains. Inter‐item correlation coefficient values ranged from .20 to .73 (Table 2).

Regarding convergent validity (Table 3), the LW‐CI scale showed strong positive correlation coefficients with DUFSS (*r*_s_ = .62) and SLS‐6 (*r*_s_ = .54).

**Table 3:** Convergent validity and internal validity of the LW‐CI scale

	LW‐CI scale					
	Domain 1: Acceptance	Domain 2: Coping	Domain 3: Self‐management	Domain 4: Integration	Domain 5: Adjustment	Total score
Convergent validity						
Age	0.00	−0.10**	−0.10**	−0.05*	−0.12**	−0.10**
Age at diagnosis	0.02	−0.06**	−0.10**	−0.03	−0.09**	−0.08**
Disease duration	−0.02	−0.06**	−0.01	−0.02	−0.06**	−0.04*
DUFSS	0.30**	0.54**	0.47	0.54	0.50**	0.62**
SLS‐6	0.40**	0.40**	0.40**	0.48**	0.50**	0.54**
Internal validity						
Domain 2: Coping	0.15	–	–	–	–	–
Domain 3: Self‐management	0.14	0.66	–	–	–	–
Domain 4: Integration	0.27	0.68	0.67	–	–	–
Domain 5: Adjustment	0.23	0.64	0.62	0.63	–	–

Abbreviations: DUFFS, Duke‐UNC Functional Social Support Questionnaire; LW‐CI scale, Living with Chronic Illness Scale; SLS‐6, Satisfaction with Life Scale.

* *p* < .05

** *p* < .01.

Regarding internal validity, correlation values for all domains of the LW‐CI scale ranged from .62 to .68, except for Domain 1: Acceptance, which showed correlation coefficients less than .30 (Table 3).

LW‐CI scale scores were significantly different for each category of PGIS (normal, mild, moderate, severe), gender and LTC (Table 4), with significantly higher scores for people with normal severity, women and individuals with hypertension (*p* < .05). Comparisons between subgroups of PGIS and type of LTC are presented in Table 4.

**Table 4:** Known groups' validity

Categories	LW‐CI scale total mean (SD)	*p*
PGIS‐based severity levels^a^		<.001^b^
Normal	75.06 (17.20)	
Mild	69.84 (17.11)	
Moderate	71.12 (18.23)	
Severe	59.73 (18.51)	
Gender		<.001
Male	68.09 (18.13)	
Female	70.83 (18.50)	
Long‐term condition		<.001^c^
Type 2 diabetes mellitus	71.53 (16.45)	
Chronic obstructive pulmonary disease	67.84 (18.86)	
Chronic heart failure	72.92 (20.14)	
Parkinson's disease	62.45 (18.57)	
Hypertension	75.20 (15.37)	
Osteoarthritis	63.19 (15.30)	

*Note*: Kruskal–Wallis test with Bonferroni correction for PGIS and long‐term condition, Mann–Whitney test for gender.

Abbreviations: LW‐CI scale, Living with Chronic Illness Scale; PGIS, Patient‐Based Global Impression of Severity Scale; SD, standard deviation.

^a^ PGIS‐based severity levels: normal, 0 points; mild, 1–2 points; moderate, 3 points; severe 4–5 points.

^b^ Except for mild–moderate comparison, not significant.

^c^ Except for Parkinson's disease with osteoarthritis, and chronic heart failure with type 2 diabetes mellitus and with hypertension comparisons, all not significant.

### 3.2 Rasch analysis

The first Rasch analysis, with all items, showed a significant model deviation: *χ* ^2^(234) = 560, *p* < .00001. Because the total scale was multidimensional (19% significant *t* tests, 95% CI: 0.165–0.215), each domain was analysed separately. Table 5 presents the goodness of fit of the LW‐CI domains, and Table 6 presents the individual item fit.

**Table 5:** Goodness of fit to the Rasch model of LW‐CI scale domains

Attribute		Criteria	Domain 1: Acceptance	Domain 2: Coping	Domain 3: Self‐management	Domain 4: Integration	Domain 5: Adjustment
Item fit residual							
Mean		0	0.067	0.545	0.441	0.634	0.371
SD		1	0.753	0.734	0.999	0.594	1.496
Person fit residual							
Mean		0	1.189	0.650	1.322	1.256	0.444
SD		1	1.730	1.224	1.544	1.490	1.362
Item–trait interaction							
*χ* ^2^(*df*)		Low	18.771 (16)	23.790 (20)	13.783 (16)	15.493 (12)	33.994 (20)
*p* Value		NS	0.281	0.251	0.615	0.216	0.0262
PSI		>0.70	0.763	0.648	0.703	0.576	0.743
Unidimensionality							
Independent *t* tests		<5%	1.33%	1.33%	0%	1.33%	3.00%
95% CI binomial		a	(−0.011, 0038)	(−0.011, 0038)	–	(−0.011, 0038)	(0.005, 0.055)

Abbreviations: CI, confidence interval; LW‐CI scale, Living with Chronic Illness Scale; NS, nonsignificant; PSI, personal separation index; SD, standard deviation.

^a^ Lower bound should be ≤0.05.

**Table 6:** Individual item fit

Item (response categories)	Location	Standard error	Fit residual	*df*	*χ* ^2^(*df* = 4)	*p* Value
Domain 1: Acceptance						
Item 1: (0–4)	0.212	0.078	−0.283	175.75	6.051	.195
Item 2: (0–3)	−1.299	0.111	0.605	175.75	6.326	.176
Item 3: (0–4)	0.312	0.079	−0.825	175.75	5.827	.212
Item 4: (0–4)	0.775	0.079	0.77	175.75	0.566	.967
Domain 2: Coping						
Item 5: (0–3)	−0.075	0.074	0.096	206.2	2.828	.587
Item 6: (0–3)	−0.072	0.075	0.048	206.2	7.006	.136
Item 7: (0–2)	0.472	0.109	1.654	206.2	9.113	.058
Item 9: (0–3)	0.038	0.073	−0.018	206.2	2.542	.637
Item 10: (0–3)	−0.362	0.076	0.948	206.2	2.301	.681
Domain 3: Self‐management						
Item 13: (0–4)	0.436	0.075	1.731	168.75	0.937	.919
Item 14: (0–4)	−0.418	0.083	−0.471	168.75	5.131	.274
Item 15: (0–4)	0.202	0.073	0.711	168.75	2.934	.569
Item 16: (0–4)	−0.220	0.08	−0.209	168.75	4.781	.311
Domain 4: Integration						
Item 17: (0–3)	−0.322	0.094	1.261	143	2.981	.561
Item 19: (0–4)	0.365	0.078	0.078	143	4.211	.378
Item 20: (0–3)	−0.043	0.096	0.564	143	8.301	.081
Domain 5: Adjustment						
Item 22: (0–3)	0.467	0.08	0.38	204.2	1.281	.865
Item 23: (0–3)	−0.235	0.082	0.775	204.2	6.801	.147
Item 24: (0–4)	−0.806	0.066	−0.553	204.2	6.175	.186
Item 25: (0–3)	0.233	0.078	−1.347	204.2	13.897	.008
Item 26: (0–3)	0.341	0.082	2.602	204.2	5.839	.212

**Figure 1:**
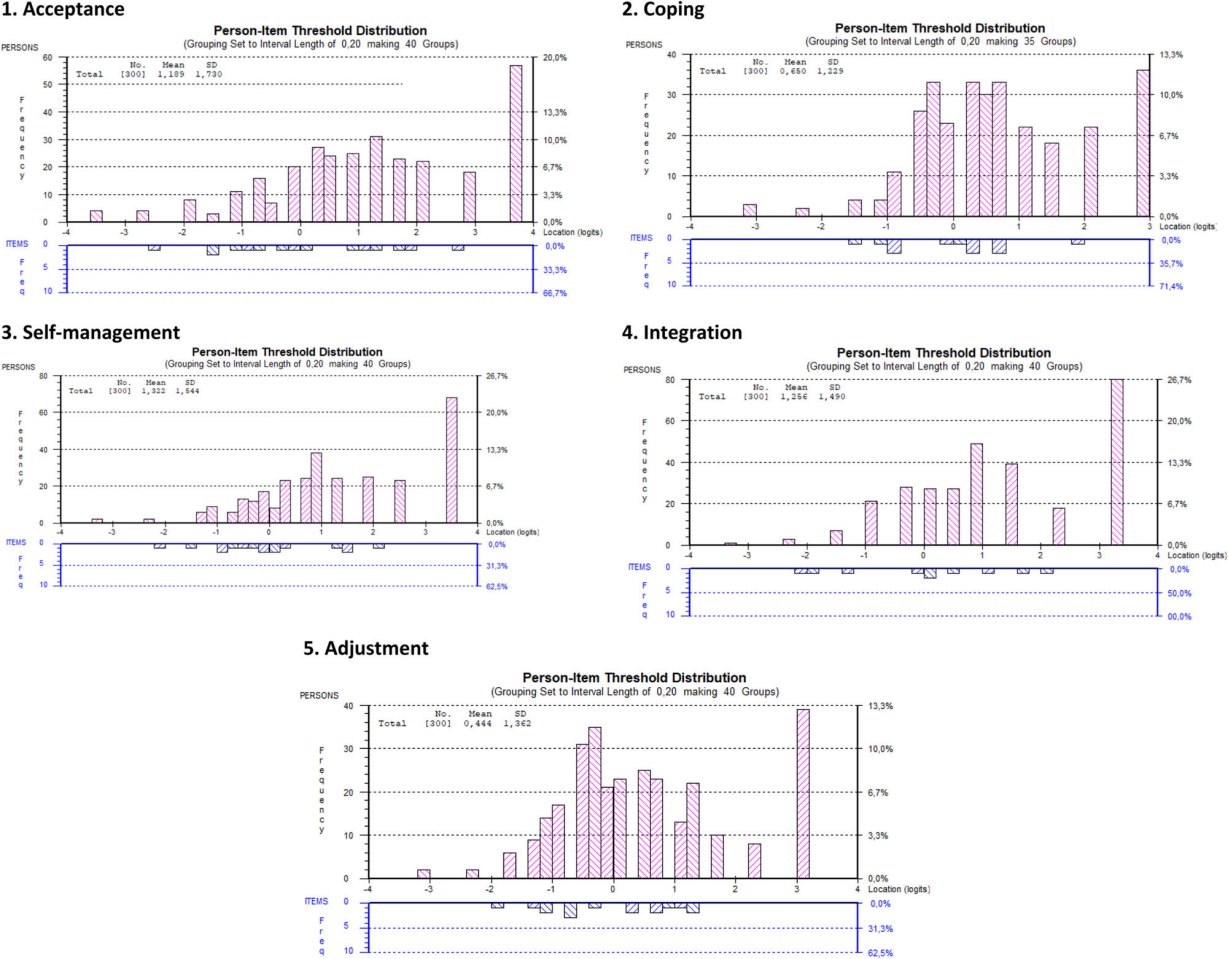
Person‐item threshold distributions for the Living with Chronic Illness domains

For the Acceptance domain, item 2 showed disordered thresholds, and categories 2–3 were collapsed. This dimension showed a good model fit, *χ* ^2^(16) = 18.772, *p* = .281, with unidimensionality, PSI = 0.763, locally independent items and absence of DIF by age, sex, disease duration or LTC. The person‐item threshold distribution shows a ceiling effect close to 20% (Figure 1).

When performing a Rasch analysis of the Coping domain, three items (2, 11 and 12) showed a significant misfit: Item 8 presented a low‐fit residual (−2.501) and items 11 and 12 presented high‐fit residuals (3.506 and 2.957, respectively). All Coping items were rescored due to disordered thresholds. The resulting scale presented an adequate fit to the Rasch model, *χ* ^2^(20) = 23.790, *p* = .252, PSI = 0.648, unidimensionality and item local independency. Item 7 presented DIF by LTC type, and item 5 showed uniform DIF by disease duration (people with a more recent diagnosis underestimated scores).

The Self‐management domain presented a good fit to the Rasch model, with ordered thresholds, unidimensionality, PSI = 0.703, locally independent items and absence of DIF: *χ* ^2^(16) = 13.783, *p* = .615.

To obtain fit in the Integration domain, the following modifications were performed: Rescoring of all items due to disordered thresholds, and deletion of items 18 (fit residual: 3.454) and 21 (fit residual: 2.848). The modified scale presented a good fit to the Rasch model, PSI = 0.576, unidimensionality and item local independency, *χ* ^2^(12) = 15.493, *p* = .216. Item 20 presented uniform DIF by LTC type. The person‐item threshold distribution showed a high (26.7%) ceiling effect (Figure 1).

In the Adjustment domain, all items except one (item 24) were rescored due to disordered thresholds. After this, the domain presented a good fit to the Rasch model, *χ* ^2^(20) = 33.994, *p* = .0262, PSI = 0.743, unidimensionality and item local independency. Although item 26 showed a marginally high‐fit residual (2.603), it was nonsignificant. The initial DIF by LTC type in items 24 and 26 cancelled out in the top–down purification procedure.

## 4 DISCUSSION

This is the first study to analyse the psychometric properties of the LW‐CI scale in a large sample of individuals with different LTCs in several Spanish‐speaking countries. Currently, the LW‐CI scale is the only PROM available in clinical practice and research that evaluates in a comprehensive manner how a person is living with an LTC.

### 4.1 Feasibility and acceptability

In general, all acceptability parameters fulfilled the standard criteria. Floor and ceiling effects could be explored using both CTT and Rasch analyses. The two domains with the highest ceiling effect at the item level, Acceptance and Self‐management, also showed a ceiling effect at the threshold level in the Rasch analysis. This could be attributed to the fact that individuals involved in this study participated voluntarily, potentially implying some degree of acceptance as well as good management of the LTC and hence, in some cases, also a positive living with the disease. A large percentage of individuals tend to score in the highest levels of these domains, which might prevent observation of changes after an intervention in people with initial high scores. To verify this result, group comparisons in the Acceptance and Self‐management domains should be performed with caution.

The quality of the data was satisfactory, exceeding 95% of computable data, due to the close supervision performed by researchers during data collection and the standardized protocol established for the data collection.

### 4.2 Reliability

Reliability was also explored using both CTT and Rasch approaches. The internal consistency of the LW‐CI scale is satisfactory, with all domains exceeding the criteria of ≥0.70 for Cronbach's *α*. However, Integration and Coping domains showed lower reliability (PSI) in Rasch analysis, which may be due to a small number of items and the ceiling effect. A lower reliability might hinder the ability of these domain scores to discriminate between two individual groups.^55^ In this sense, further studies are recommended to verify the reliability of both domains.

### 4.3 Convergent validity

Regarding convergent validity, the results corroborate the strong relationship between living with an LTC and perceived social support of the person as well as satisfaction with life. Living with an LTC presents low correlations with disease‐related factors, namely, duration. Congruently, all items except one were unbiased by disease duration in Rasch analysis. Further studies are needed to confirm whether DIF by duration in item 5 (I try to cope and fight the disease) remains, as it was not identified in a study with a PD population.^17^ In addition, two items showed bias by LTC type: Coping item 7 (I try to see the positive side of the disease) and Integration item 20 (Although I have the disease, I feel satisfied with my life). In item 20, people with hypertension tended to overestimate scores, whereas for item 7, there was no a clear pattern. Further studies are needed to confirm these DIF results, and the results at the item level should be interpreted with caution when comparing individuals. However, these two items represent less than 10% of the scale. Even though a previous study had found that one item was biased by age, we did not replicate this finding in the present study, showing the absence of DIF by age for all items.^17^ Therefore, the LW‐CI scale could be useful across a general adult (≥18 years) population living with at least one LTC in clinical practice.

Convergent validity findings were also identified in previous validation studies^16^, ^18^, ^19^, ^20^, ^21^ and additionally were confirmed through linear regression models performed with an LTC population such as PD.^5^ According to these results, there is clearly a need to place the emphasis on the person and in his/her daily living with the condition, and not just on the disease. Each person with an LTC must be seen as a unique person, independent of the stage or the severity of the disease. Therefore, it is necessary to incorporate multidisciplinary and individualized interventions in current health and social services, focusing on the factors that directly influence living with an LTC, namely, social support and satisfaction with life. Consequently, possible negative aspects of the daily living with an LTC, such as lack of support, loneliness or dissatisfaction with life, could be prevented, and a more positive living can be achieved. In this sense, person‐centred interventions or individualized health and social pathways could be implemented in clinical practice, incorporating nonpharmacological or disease‐specific measures that address the factors that are paramount in the daily living with an LTC. Therefore, LTC programmes that mobilize and optimize the use of community resources and increase social networks and support seem to be the direction for the management of LTCs.

### 4.4 Internal validity

The internal validity for LW‐CI scale domains was excellent, except for Domain 1: Acceptance, with correlation coefficients under .30 with the rest of the domains. This result is consistent with previous validation studies^16^ and conceptual work^4^ showing that Acceptance is always the first domain to achieve a positive living. Only when the person has accepted and assumed his/her illness can he or she move on to the other domains, such as Coping or Self‐management. Therefore, according to the poor correlation that Acceptance showed with other dimensions of the LW‐CI scale and based on the aforementioned conceptual framework, these findings were expected because Acceptance is considered an internal, illness‐independent, process through which the person recognizes and accepts the reality. Interval validity was also supported by Rasch analysis, showing that each domain is unidimensional, and providing support for the calculation of domain scores.

### 4.5 Known groups' validity

The LW‐CI scale demonstrated satisfactory known groups' validity, yielding significantly different scores between men and women, patient‐based global impression of disease severity and LTC type. The result related to differences among types of LTC is understandable, as each condition has its particular symptoms and evolution. For example, PD is defined as a complex and disabling disorder characterized by being a neurodegenerative and progressive disorder, while hypertension is a cardiovascular condition that could be managed with healthy lifestyle patterns. In this sense, the individual may have a better or worst process based on the LTC characteristics. However, further studies are suggested to verify this result. Regarding gender differences, existing evidence also justifies the identified results. For example, Crispino et al.^56^ stated that in general, women with PD showed more positive disease outcomes than men. Besides, other studies performed in individuals with T2DM^57^ showed that women with T2DM are at greater risk than men of psychosocial maladjustment, a poorer cardiovascular profile and/or noncompliance with treatment goals. The observed significant differences by gender are not due to a bias, as no item presented DIF by gender. However, the significantly lower scores in hypertension individuals should be interpreted with caution, since two items were biased by LTC type. Therefore, LW‐CI scores may be interpreted similarly across LTC types, thus allowing group comparisons, except for two items.

### 4.6 Rasch analysis

To achieve model fit in Rasch analysis, some modifications were performed. First, the response scale was simplified for some items, similar to a previous study with only a PD population.^17^ This result suggests that individuals have difficulty in setting apart from some response options and that the response scale should be codified differently than what was initially proposed. This does not modify the scale administration. Another modification suggested by Rasch analysis was the deletion of certain items. Again, they might be maintained in the scale administration, as they can provide useful clinical information. However, when calculating linear scores, these items should not be considered, as they are either redundant or they measure a different construct.^26^ In both the present study and the previous study,^17^ item 8 was found to be redundant, and item 18 measured a different construct.

Recently, other measures have been designed and validated as the Long‐Term Conditions Questionnaire^10^ with Rasch analysis^58^ in a wide and representative sample of people living with LTCs in three regions of England with the general aim of evaluating how people live/manage their LTC. However, despite the potential relevance of the Long‐Term Conditions Questionnaire, this scale seems to evaluate quality‐of‐life outcomes instead of living with an LTC and excludes aspects related to acceptance, which is an essential attribute when living with an LTC.^4^ Moreover, currently, there are multiple measuring scales that seem to evaluate the phenomena of living with an LTC.^9^ However, existing scales evaluate the process in a fragmented way by measuring some elements of the process or related processes, such as quality of life.^9^ Consequently, to our knowledge, the LW‐CI scale could be considered the only available person‐centred measure that evaluates the complex process of living with an LTC in a comprehensive manner. Using this scale, healthcare professionals could identify aspect/s of the process that the patient finds more challenging and consequently, referrals, interventions or signposting to services or resources could take place based on the assessment and identified needs. For example, the scale itself could be part of interventions through which patients could complete the scale at home, increasing their awareness of how they live with the LTCs and in their annual or periodic review with the GP, nurse or specialist, the results from the assessment could also be discussed for further support. This could be equally applied to research projects. At present, a cross‐culturally adapted version of the scale has been produced in the United Kingdom (called the living with long‐term condition scale)^59^ and full validation study is ongoing, which would allow health and social care professionals to implement person‐centred care pathways and referral processes.

This validation study is novel because the LW‐CI scale is the first validated rating scale for assessing the phenomenon of living with an LTC. Moreover, this scale has been used for the first time to assess several highly prevalent LTCs in different Spanish‐speaking countries using two complementary analytical approaches to ascertain its psychometric properties. Therefore, considering the results, the LW‐CI scale could be used in clinical practice to evaluate the degree of living with several highly prevalent LTCs in different Spanish‐speaking countries.

### 4.7 Limitations

We acknowledge that the limitations of the study are mainly related to the convenience sample, with a heterogeneous representation of LTCs and countries. Additionally, although country‐based samples were not large enough to produce any country‐based (cultural) analysis, further international studies are needed to test item bias by country as well as cultural differences within countries. Besides, considering the sample diversity, including people from six different countries, sociodemographic data related to ethnicity were not collected. Additionally, for this validation study patient and public involvement (PPI) was not conducted because the aim of this study was to statistically analyze the psychometric properties of the LW‐CI scale. However, PPI was a key aspect during the development of the scale and piloting phase before the main validation studies. Finally, data collection was performed at different times for PD and the rest of the LTCs. However, to ensure homogeneity of the process and avoid errors, a clear and standardized protocol was followed, and data quality was equally valid for the purpose of this study. On the other hand, the strengths of this study are related to the sample diversity, including the highly prevalent and prototypical LTC population as well as the large age range population, which led to the real value of the LW‐CI scale psychometric properties for a general LTC population. Finally, the combined application of the classic test theory approach and Rasch analysis is highly recommended for evaluating patient outcome report measures such as the LW‐CI scale.

## 5 CONCLUSION

This study fulfils an important gap in the literature regarding person‐centred measures to evaluate the process of living with an LTC. After some adjustments, the LW‐CI domains showed a good fit to the Rasch model, with unidimensionality, item local independency and moderate reliability, providing scores on a true interval scale. This last feature is key for using the scale in clinical trials. Except for two items, the LW‐CI scale was free from bias by LTC type. In this sense, although cautious use is suggested, the LW‐CI scale is ready for use in clinical practice and research in Spanish‐speaking populations living with different LTCs, which could lead to more person‐centred care and individualized psychosocial interventions. Therefore, future implementation studies are suggested to assess the usefulness of the LW‐CI scale in clinical practice.

## CONFLICT OF INTERESTS

The authors declare that there are no conflict of interests.

## AUTHOR CONTRIBUTIONS

Carmen Rodriguez‐Blazquez, Maria João Forjaz, and Alba Ayala: *design of the work, data analysis, interpretation of the findings, drafting the article, critical revision and substantial contribution of the article, approval of the final version for publication*. Mari Carmen Portillo: *conception and design of the work, expert consulting, critical revision and substantial contribution of the article, approval of the final version for publication*. Leire Ambrosio: *principal author of the scale, principal investigator of the project and responsible for funding of the project, conception and design of the work, oversight of the project, data collection, interpretation of the findings, drafting the article, critical revision and substantial contribution of the article, approval of the final version for publication*. The corresponding author, in representation of the rest of the undersigning individuals, guarantees the precision, transparency and honesty of the data and information contained in the study and additionally that none of the relevant information has been omitted, and that all discrepancies among the authors have been adequately resolved and described.

## Supporting information

Supporting information.

## Data Availability

The data that support the findings of this study are available on request from the corresponding author. The data are not publicly available due to privacy or ethical restrictions.
