# Supplementary material for: Living with Chronic Illness Scale: International validation through the classic test theory and Rasch analysis among Spanish‐speaking populations with long‐term conditions
Source: Health Expect. 2021 Sep 7;24(6):2065–77. doi: 10.1111/hex.13351 (PMC8628594; doi:10.1111/hex.13351)
Supplement: Supplementary file 1 — Supporting information. [file HEX-24-2065-s001.docx]

**Appendix. Members of the LW-CI scale (in Spanish EC-PC) Validation Group:**

Mario Alvarez: Movement Disorder and Biostatistics Units, Neurological Service, Carlos Andrade Marín Hospital, Quito, Ecuador. Leire Ambrosio: School of Health Sciences, NIHR ARC Wessex. University of Southampton, Southampton, United Kingdom. Tomoko Arakaki: Department of Neurology, JM Ramos Mejia Hospital, Buenos Aires, Argentina. Marta Aranda-Gallardo: Department of Internal Medicine, Costa del Sol Hospital, Malaga, Spain. Alba Ayala: National School of Public Health, Institute of Health Carlos III and REDISSEC, Madrid, Spain. Victor Campos Arillo: Vithas-Xanit International Hospital, Malaga, Spain. Neus Caparros: Law Department, La Rioja University, La Rioja, Spain. Jorge Caro-Bautista: Andalusian Public Health System, District of Primary Health Care of Málaga-Valle del Guadalhorce, Malaga, Spain. Ana Carvajal: Faculty of Nursing. University of Navarra, Navarra, Spain. Gloria Carvajal: Faculty of Nursing and Rehabilitation, Universidad de La Sabana, Chía, Colombia. Silvia Corchon: Faculty of Nursing and Chiropody, University of Valencia, Valencia, Spain. Nerea Elizondo: Navarra Healthcare System, Navarra, Spain. Maria João Forjaz: National Centre of Epidemiology, Institute of Health Carlos III and CIBERNED, Madrid, Spain. Alejandra Fuentes-Ramirez: Faculty of Nursing and Rehabilitation, Universidad de La Sabana, Chía, Colombia. Nélida Susana Garretto: Department of Neurology, JM Ramos Mejia Hospital, Buenos Aires, Argentina. Ivonne Pedroso Ibañez: Department of Movement Disorders and Neurodegeneration, La Habana, Cuba. Lorena Lopez: Madrid Healthcare System, Madrid, Spain. Juan Carlos Martinez Castrillo, Ramon y Cajal University Hospital, Madrid, Spain. Pablo Martinez-Martin: National Centre of Epidemiology and CIBERNED. Carlos III Institute of Health, Madrid, Spain. Alfonso Meneses: Faculty of Nursing, Physiotherapy and Podiatry, University Complutense of Madrid, Madrid, Spain. Maria Victoria Navarta-Sanchez: Faculty of Medicine, Autonomous University of Madrid, Madrid, Spain. David Perez-Manchon: Faculty Health, Camilo Jose Cela University, Madrid, Spain. Mari Carmen Portillo: School of Health Sciences, NIHR ARC Wessex. University of Southampton, Southampton, United Kingdom. Carmen Rodriguez-Blazquez: National Centre of Epidemiology and CIBERNED, Carlos III Institute of Health, Madrid, Spain. Mayela Rodriguez-Violante, National Institute of Neurology and Neurosurgery, Movement Disorders Clinic, Mexico City, Mexico. Manuel Ignacio Ruiz de Ocenda: La Rioja Health Care System, La Rioja, Spain. Marcos Serrano Dueñas: Movement Disorder and Biostatistics Units, Neurological Service, Carlos Andrade Marín Hospital, Quito, Ecuador. Eva Timonet: Department of Cardiology, Costa del Sol Hospital, Malaga, Spain. Maria Eugenia Ursua: Navarra Healthcare System, Navarra, Spain.
